# Synthetic Bacterial Community of Duckweed: A Simple and Stable System to Study Plant-microbe Interactions

**DOI:** 10.1264/jsme2.ME20112

**Published:** 2020-12-02

**Authors:** Hidehiro Ishizawa, Minami Tada, Masashi Kuroda, Daisuke Inoue, Hiroyuki Futamata, Michihiko Ike

**Affiliations:** 1 Division of Sustainable Energy and Environmental Engineering, Graduate School of Engineering, Osaka University, 2–1 Yamadaoka, Suita, Osaka 565–0871, Japan; 2 Research Institute of Green Science and Technology, Shizuoka University, 3–5–1 Johoku, Naka-ku, Hamamatsu, Shizuoka, 432–8561, Japan; 3 Faculty of Social and Environmental Studies, Tokoha University, 6–1 Yayoi-cho, Suruga-Ku, Shizuoka, Shizuoka, 422–8581, Japan

**Keywords:** synthetic ecology, duckweed, plant-microbe interaction, SynCom, bacterial community assembly

## Abstract

A complete understanding of the plant microbiome has not yet been achieved due to its complexity and temporal shifts in the community structure. To overcome these issues, we created a synthetic bacterial community of the aquatic plant, duckweed. The synthetic community established with six bacterial strains showed a stable composition for 50 days, which may have been because duckweed maintains a similar physiological status through its clonal reproduction. Additionally, the synthetic community reflected the taxonomic structure of the natural duckweed microbiome at the family level. These results suggest the potential of a duckweed-based synthetic community as a useful model system for examining the community assembly mechanisms of the plant microbiome.

Plants form taxonomically-structured microbiomes; however, the mechanisms underlying community assembly have not yet been elucidated by microbiome research. The use of synthetic communities, *i.e.* a mixture of isolated strains mimicking the natural microbiome, recently emerged as a novel experimental approach. The merits of using synthetic communities include their simplicity, reproducibility, and ease of detecting causality (Castrillo *et al.*, 2017; Vorholt *et al.*, 2017; Durán *et al.*, 2018; Herrera Paredes *et al.*, 2018; Liu *et al.*, 2019). Previous studies that utilized plant-associated synthetic communities (often referred to as SynCom) provided high-confidence data on the mechanisms by which specific genes and metabolites influence microbial community assembly (Bodenhausen *et al.*, 2014; Lebeis *et al.*, 2015; Bai *et al.*, 2015; Carlström *et al.*, 2019; Voges *et al.*, 2019). Niu *et al.* (2017) created a seven-membered synthetic bacterial community of maize roots, and investigated the mechanisms by which bacterial interspecies interactions affect community assembly.

We herein report the establishment of a synthetic bacterial community associated with the aquatic plant, duckweed. Duckweed is an attractive model plant for examining plant-microbe interactions due to its easy gnotobiotic cultivation and manipulation (Appenroth *et al.*, 2016; Yamakawa *et al.*, 2018; Ishizawa *et al.*, 2019). More importantly, duckweed populations maintain a certain age structure and physiological status over long periods of time through their budding-based growth (Datko *et al.*, 1980), whereas most plants markedly change their morphology, physiology, and associated microbiome with development and senescence (Zhalnina *et al.*, 2018; Hara *et al.*, 2019). Therefore, we hypothesized that the first “stable” synthetic community, which maintains similar properties irrespective of the culture time, may be established with duckweed. Since current analytical technologies for genes and metabolites provide only a “snapshot” information at the time of measurement, temporal shifts in plant and microbial physiologies have been a major obstacle to detailed analyses. Therefore, a stable synthetic system may facilitate clearer investigations of plant-microbe interactions, leading to unique insights in this field.

In the present study, we initially grew duckweed in a natural pond environment and isolated duckweed-associated bacteria as candidate members of the synthetic community. A laboratory stock of sterilized duckweed (*Lemna minor* RDSC5512), which was previously sterilized (Suzuki *et al.*, 2014) and routinely cultured with modified Hoagland (MH) medium (Toyama *et al.*, 2006) in a growth chamber (28°C, photon flux of 80‍ ‍μmol m^–2^ s^–1^, 16-h light cycle), was floated on the Inukai pond (Suita, Osaka, Japan) within a stainless steel mesh box (20×20×20‍ ‍cm, opening of 1×1‍ ‍mm) from October 8th to 18th, 2018. Approximately 1,000 fronds were transplanted, and 50 were collected after 1, 3, 5, 7, and 10 days and then preserved at –80°C until DNA extraction. Another 50 fronds were collected after 7 days as the isolation source of co-existing bacteria.

A culture-independent analysis of the duckweed microbiome was performed as described previously (Ishizawa *et al.*, 2020). Briefly, the DNA of epiphytic bacteria was extracted using the Cica Genius DNA extraction kit (Kanto Chemical) and the V4 region of the bacterial 16S rRNA gene was amplified with two-step PCR using 5′-tailed primers (515F–805R; Caporaso *et al.*, 2010) and Illumina index PCR primers (Illumina). Multiplex sequencing was performed on an Illumina Miseq platform (300-bp paired-end).

Raw sequence reads were demultiplexed in Qiime v1.9.1. Quality filtering, the merging of paired-end reads, chimera removal, singleton removal, and taxonomy assignments were performed with dada2 (Callahan *et al.*, 2016) in R v3.5.2. Forward and reverse sequences were trimmed from 20 to 280 bp and from 20 to 160 bp, respectively. The SILVA database (v138; Quast *et al.*, 2013) was used for the taxonomic assignment of amplicon sequence variants (ASVs). The relative abundance of bacterial taxa was estimated with 35,386–40,233 reads per sample after removing non-prokaryotic ASVs. The Shannon index was calculated using the “vegan” package in R.

Regarding bacterial isolation, fresh plant samples (50 fronds) were gently washed with *ca.* 40‍ ‍mL of sterile MH medium and homogenized with a disposable homogenizer (Biomasher II; Nippi) until no large plant tissues (>*ca.* 0.5‍ ‍mm) remained. The homogenate was diluted and spread on R2A (Daigo, Nihon Pharmaceutical), tryptic soy broth (TSB; Difco), 1/100 R2A, or 1/100 TSB medium with 1.5% agar or 1.0% gellan gum (with 0.3‍ ‍g L^–1^ MgSO_4_ as the cation source) and 100‍ ‍mg L^–1^ cycloheximide. Morphologically distinct colonies were exhaustively selected, purified, and preserved at –80°C with 15% glycerol for further use. Regarding taxonomic identification, partial 16S rRNA gene sequences (primers 27F–1392R) were amplified and sequenced as described previously (Ishizawa *et al.*, 2017). The sequences obtained were compared to those in the GenBank/EMBL/DDBJ databases using BLAST. The neighbor-joining phylogenetic trees of the isolates were constructed using MEGA7 software v7.0.14 with dominant ASVs in the pond-grown duckweed microbiome.

The synthetic bacterial community was constructed by co-culturing duckweed with 16, 6, or 5 bacterial isolates. Each isolate was cultivated overnight (28°C, 120 rpm) in 10‍ ‍mL of R2A medium (supplemented with 2% methanol for *Methylophilaceae* strains) in a vial, pelleted (10,000×*g*, 4°C, 5‍ ‍min), re-suspended three times in 5‍ ‍mL of sterile MH medium, and then mixed at approximately the same cell densities based on the optical density at 600 nm (OD_600_). The mixture with a known OD_600_ was added to 60‍ ‍mL MH medium in the flask to adjust the final OD_600_ to *ca.* 0.0005. Ten fronds of sterile *L. minor* were transplanted into the flask to initiate the co-cultivation under controlled conditions (28°C, 80‍ ‍μmol m^–2^ s^–1^, 16-h light cycle). Under our growth conditions, duckweed grew exponentially to *ca.* 50–60 fronds in 5 days. Hence, the synthetic system was maintained by transplanting ten duckweed fronds (with bacteria attached) into new medium every 5 days. In the experiments using 16- and 5-membered communities, plant samples for the DNA analysis were collected at the end of the second batch cultivations. In the six-membered community, samples were collected from each of the triplicate flasks after the 1st, 2nd, 5th, and 10th batch cultivations. We also estimated the total bacterial colonization density (CFU per frond) at each sampling point by plating diluted homogenates of plant samples (from each flask) on R2A agar supplemented with 2% methanol. The number of colonies was counted after a 3-day incubation at 28°C.

The relative abundance of bacterial strains in the synthetic communities was evaluated by Illumina Miseq sequencing as described above, and the V5-V6 region of the bacterial 16S rRNA gene (primers 799F–1185mR) (Chelius and Triplett, 2001; Hodkinson and Lutzoni, 2009) was analyzed to distinguish all bacterial strains. Reads of ASVs with sequences that perfectly matched that of or had a single base mismatch from the inoculated strains (43,484–56,546 reads per sample) were used to calculate relative abundance. The family level composition of the synthetic communities was compared to that of the natural duckweed microbiome and other plant-associated microbiomes sequenced in previous studies (Schlaeppi *et al.*, 2014; Xie *et al.*, 2015; Agler *et al.*, 2016; Niu *et al.*, 2017; Toju *et al.*, 2019; Acosta *et al.*, 2020; Huang *et al.*, 2020; Ishizawa *et al.*, 2020). Renkonnen dissimilarity (1 – the Renkonnen similarity index) was used for comparisons because this metric is robust against differences in sample sizes and diversity (Wolda, 1981). Non-metric multidimensional scaling (NMDS) was performed for visualization using the “vegan” package in R.

Figure 1 shows the bacterial community composition associated with the pond-grown duckweed. Duckweed assembled a unique microbial community within 1 day, and the dominant bacterial families (>2% relative abundance on average, shown in Fig. 1) remained unchanged throughout the culture period. These dominant families are consistent with previous findings on the duckweed microbiome (Xie *et al.*, 2015; Acosta *et al.*, 2020; Huang *et al.*, 2020; Ishizawa *et al.*, 2020; Iwano *et al.*, 2020; Iwashita *et al.*, 2020), indicating that duckweed attracts specific bacterial taxa irrespective of the environmental context. On the other hand, the dominant genera or ASVs within each family were repeatedly replaced over 10 days (Fig. S1). Hence, the composition at finer phylogenetic levels may be determined by rather stochastic processes.

Given the consistent family-level composition of the duckweed microbiome, we attempted to establish a synthetic community with a similar family-level structure to natural communities. To achieve this, we selected 16 bacterial isolates belonging to the six dominant families (*Caulobacteraceae*, *Comamonadaceae*, *Flavobacteriaceae*, *Methylophilaceae*, *Oxalobacteraceae*, and *Sphingomonadaceae*) for further experiments (Table S1, Fig. S2, S3, S4, S5, S6, and S7). These families were selected based on their common presence at relatively high abundance in the *L. minor* microbiome. Collectively, they accounted for 44.1–58.5% of the pond-grown duckweed microbiome analyzed in the present study (Fig. 1), and on average 78.3% of that previously observed for the same *L. minor* clone (Ishizawa *et al.*, 2020).

When we inoculated sterile duckweed with the 16 bacterial strains (2–3 strains per family), all six families co-existed at a certain abundance (2.0–23.5%), whereas the four strains (DW043, DW096, DW159, and DW160) failed to survive in the community (<0.1%) (Fig. 2A). Moreover, only a single strain appeared to thrive from each of the six families. These results suggest that bacterial strains in the same family are more likely to compete for similar niches, while strains in different families co-exist due to the different niche preference (Martiny *et al.*, 2015; Goldford *et al.*, 2018).

We then constructed the synthetic community with six bacterial strains selected from the 16-membered community (DW039, DW067, DW100, DW102, DW145, and DW155) for further simplification, and found similar communities among triplicates throughout the 50-day cultivation period (Fig. 2B). The bacterial colonization density was also maintained at between 10^6^ to 10^7^ CFU per frond for 50 days (Table S2). This high stability over 50 days was outstanding because the plant microbiome inevitably exhibits time-dependent community shifts with host development and senescence (Sugiyama *et al.*, 2014; Xu *et al.*, 2018; Zhalnina *et al.*, 2018; Hara *et al.*, 2019). The synthetic community of maize roots established by Niu *et al.* (2017) also showed a dynamic community shift during their 15-day monitoring period. Therefore, as our initial expectation, a static synthetic community was established, which appeared to be aided by the unchanging physiological status of duckweed. In addition, the synthetic community reflected the taxonomic profile of the natural duckweed microbiome, in that *Caulobacteraceae*, *Comamonadaceae*, and *Methylophilaceae* were the most dominant and the other three families co-existed at a specific relative abundance (>0.3%). The results of the comparative analysis indicated that our synthetic community structure at the bacterial family level reflected the unique structure of the natural duckweed microbiome (Fig. 3A), and was the most similar to those associated with the same *L. minor* clone grown under the same light, thermal, and nutrient conditions (Ishizawa *et al.*, 2020; Fig. 3B).

We also performed drop-out experiments on the six-membered community. When strong interactions (*e.g.* competition or metabolite exchange) exist among the six strains, the deletion of a single strain is expected to impact the community structure of the remaining members. Similar experiments on maize, alfalfa, and *Arabidopsis*-based synthetic communities identified some keystone species that conspicuously distorted the remaining community upon deletion (Niu *et al.*, 2017; Carlström *et al.*, 2019; Moccia *et al.*, 2020). However, in the present study, all six drop-out communities showed similar structures to those expected from the original community (Fig. 2C). Nevertheless, one of the three dominant strains (DW039, DW102, and DW145) specifically increased upon the deletion of another dominant strain, suggesting that multiple ecological processes, including niche segregation and interspecies interactions, are involved in the formation of our synthetic community.

In conclusion, we herein established a synthetic bacterial community of duckweed that maintained its community structure irrespective of the culture time. This high stability is a unique feature of our synthetic system that will enable a more detailed understanding of plant-microbe interactions with existing technologies. The community assembly of the plant microbiome is currently described as a two-step selection model, in which plant-derived substrates induce the initial taxonomic selection, and undefined genotypic factors fine-tune the co-existing members (Bulgarelli *et al.*, 2013). Goldford *et al.* (2018) recently postulated that substrate-driven selection mainly occurs for functions conserved at the bacterial family level. Based on these concepts, the assembly mechanisms of the duckweed microbiome warrant further study from two aspects: (i) the functions of the dominant families that make them competent in the duckweed microbiome and (ii) the factors influencing the winners of within-family competition. Future analyses on bacterial functions and interactions in the duckweed-based synthetic community will contribute to a more detailed understanding of the plant microbiome.

All raw sequence data related to the present study are available in the DDBJ Sequence Read Archive under accession numbers DRA010591 and LC573430–LC573445.

## Citation

Ishizawa, H., Tada, M., Kuroda, M., Inoue, D., Futamata, H., and Ike, M. (2020) Synthetic Bacterial Community of Duckweed: A Simple and Stable System to Study Plant-microbe Interactions. *Microbes Environ ***35**: ME20112.

https://doi.org/10.1264/jsme2.ME20112

## Supplementary Material

Supplementary Material

## Figures and Tables

**Fig. 1.**
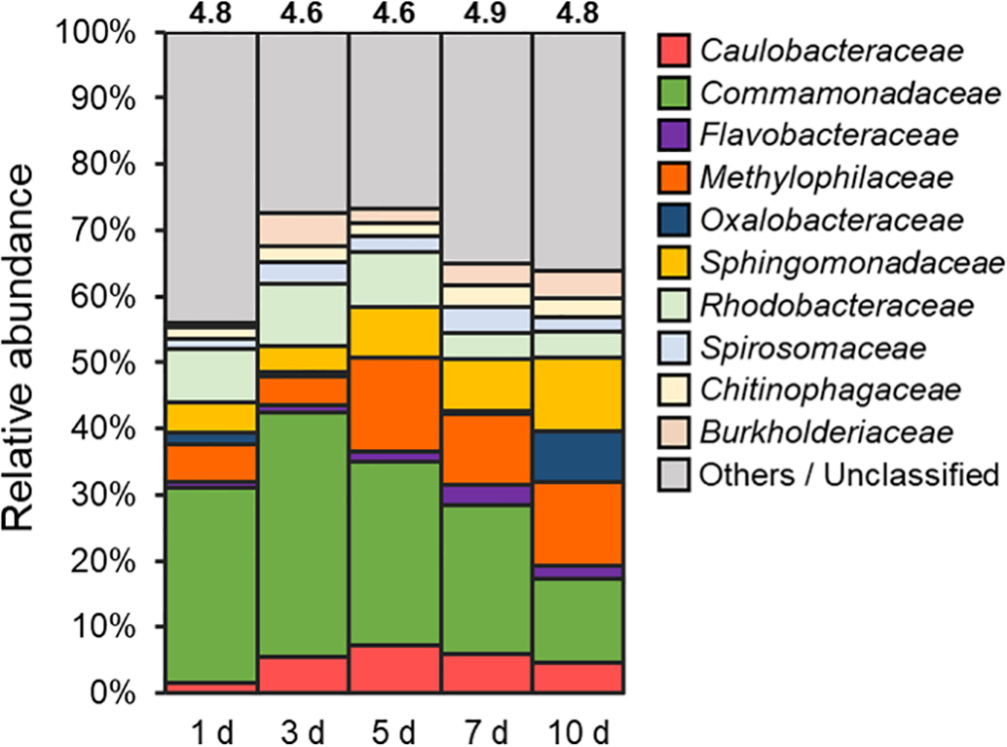
Phylogenetic distribution of duckweed-associated bacterial communities in the natural pond environment. *Lemna minor* plants were grown in the Inukai pond and the epiphytic bacterial community was analyzed at various times. Values above the bars indicate the alpha diversity (Shannon index). Families with average relative abundance <2.0% were assembled as “Others”.

**Fig. 2.**
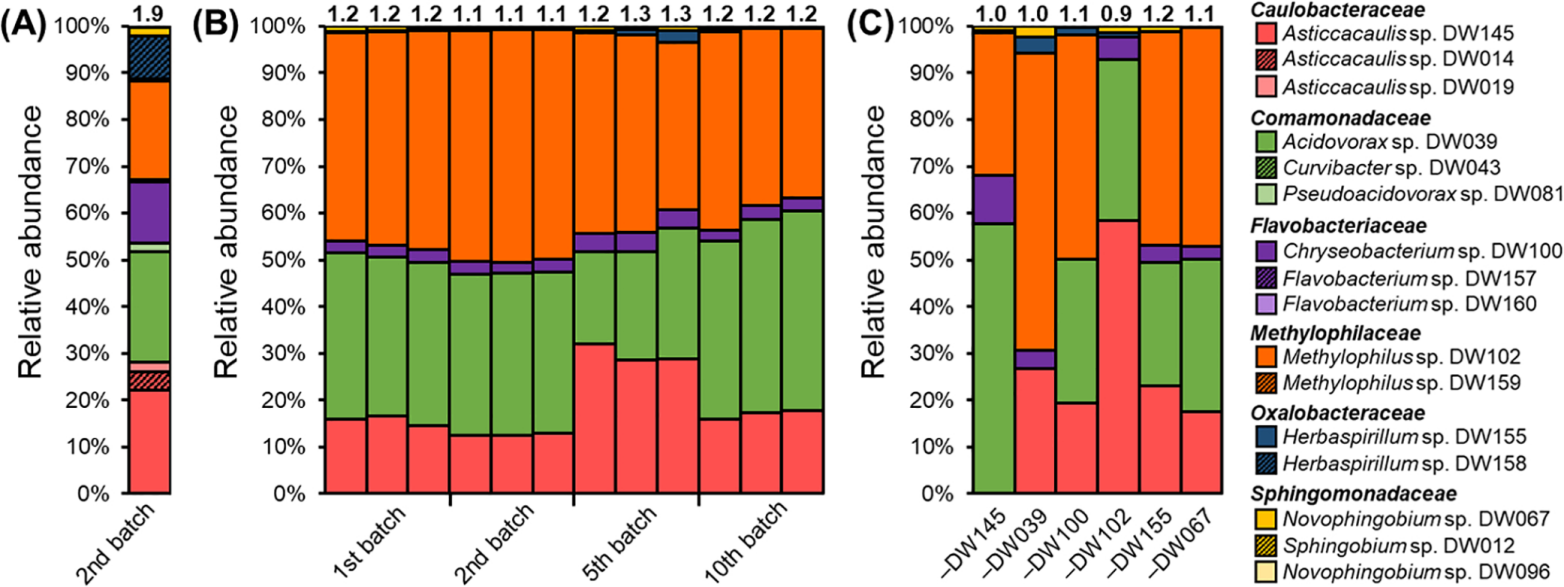
Relative abundance of bacterial strains in synthetic communities of *Lemna minor*. (A) The 16-membered synthetic community analyzed after the 2nd batch (10 days). (B) The six-membered synthetic community monitored up to the 10th batch (50 days). (C) Drop-out communities of the six-membered synthetic community analyzed after the 2nd batch (10 days). Values above the bars indicate the alpha diversity (Shannon index).

**Fig. 3.**
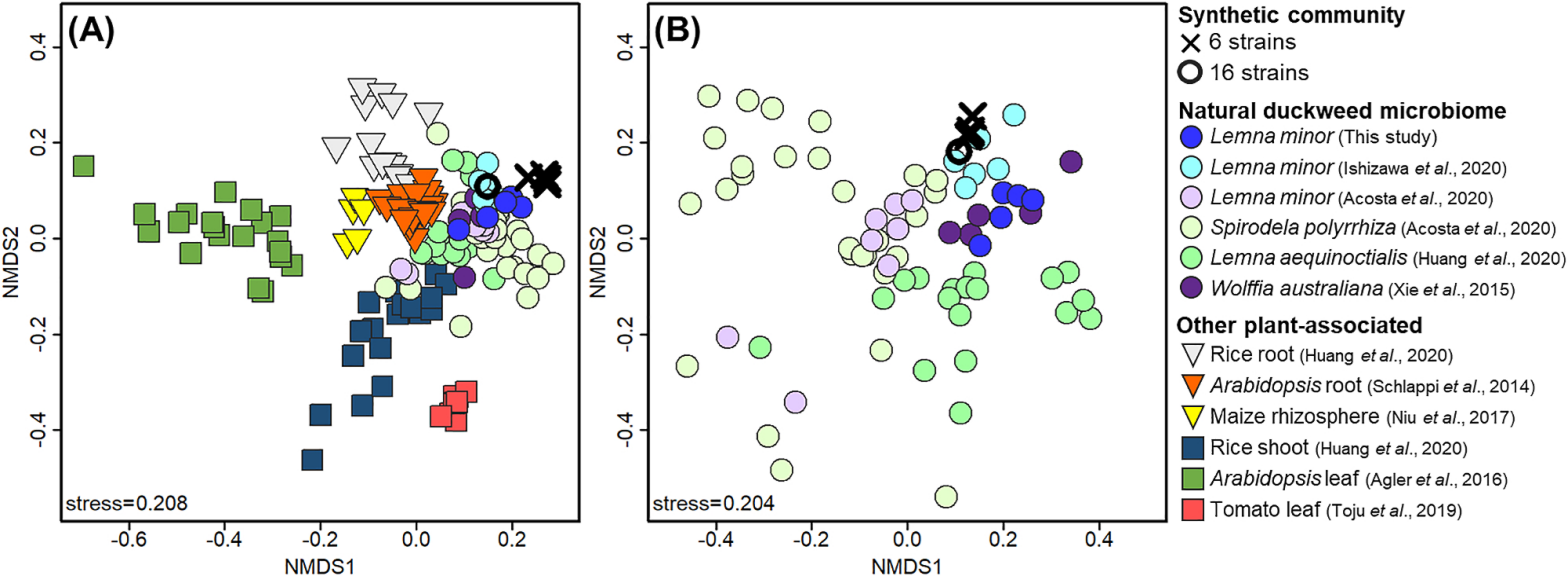
Comparison of taxonomic profiles of synthetic communities with several plant-associated microbiomes (A) and natural duckweed-associated microbiomes (B). The first two dimensions of a non-metric multidimensional scaling analysis were plotted based on Renkonnen dissimilarity at the bacterial family level.
