# Supplementary material for: Synthetic Bacterial Community of Duckweed: A Simple and Stable System to Study Plant-microbe Interactions
Source: Microbes Environ. 2020 Dec 2;35(4):ME20112. doi: 10.1264/jsme2.ME20112 (PMC7734406; doi:10.1264/jsme2.ME20112)
Supplement: Supplementary file 1 — Supplementary Material [file 35_20112_s1.pdf]

Table S1 List of bacterial strains used to construct synthetic bacterial communities.

Strain	Family	Isolation medium	Closet match in the Blast search (Accession number, percent identity)	Compared length (bp)	Accession number
DW014	Caulobacteraceae	R2A gellan gum + cycloheximide	*Asticcacaulis excentricus* CB_48 (NR_114730, 99.92%)	1,186	LC573430
DW019	Caulobacteraceae	R2A agar + cycloheximide	*Asticcacaulis excentricus* CB_48 (NR_114730, 99.92%)	1,205	LC573431
DW145	Caulobacteraceae	1/100 TSB gellan gum	*Asticcacaulis excentricus* CB_48 (NR_114730, 98.97%)	1,165	LC573432
DW039	Comamonadaceae	1/100 R2A agar + cycloheximide	*Acidovorax temperans* PHL (NR_028715, 99.24%)	1,186	LC573433
DW043	Comamonadaceae	1/100 TSB gellan gum	*Curvibacter delicatus* 146 (NR_028713, 98.23%)	1,189	LC573434
DW081	Comamonadaceae	1/100 TSB gellan gum	*Pseudacidovorax intermedius* CC-21 (NR_044241, 99.92%)	1,250	LC573435
DW100	Flavobacteriaceae	R2A gellan gum + cycloheximide	*Chryseobacterium gambrini* 5-1St1a (NR_042505, 99.75%)	1,199	LC573436
DW157	Flavobacteriaceae	R2A agar + cycloheximide	*Flavobacterium shanxiense* YF-2 (NR_169385, 99.45%)	1,266	LC573437
DW160	Flavobacteriaceae	R2A agar + cycloheximide	*Flavobacterium daejeonense* GH1-10 (NR_043654, 97.39%)	1,263	LC573438
DW102	Methylophilaceae	1/100 TSB agar	*Methylophilus rhizosphaerae* CBMB127 (NR_116239, 99.15%)	1,287	LC573439
DW159	Methylophilaceae	R2A agar + cycloheximide	*Methylophilus rhizosphaerae* CBMB127 (NR_116239, 99.34%)	1,216	LC573440
DW155	Oxalobacteraceae	1/100 TSB agar	*Herbaspirillum seropedicae* Z67 (NR_114720, 99.48%)	1,161	LC573441
DW158	Oxalobacteraceae	R2A agar + cycloheximide	*Herbaspirillum aquaticum* IEH 4430 (NR_116605, 99.77%)	1,278	LC573442
DW067	Sphingomonadaceae	R2A gellan gum + cycloheximide	*Novosphingobium bradum* STM-24 (NR_153728, 97.76%)	1,203	LC573443
DW012	Sphingomonadaceae	R2A gellan gum + cycloheximide	*Sphingomonas wittichii* RW1 (NR_074268, 98.39%)	1,241	LC573444
DW096	Sphingomonadaceae	R2A agar + cycloheximide	*Novosphingobium capsulatum* NBRC12533 (NR_113591, 99.35%)	1,232	LC573445

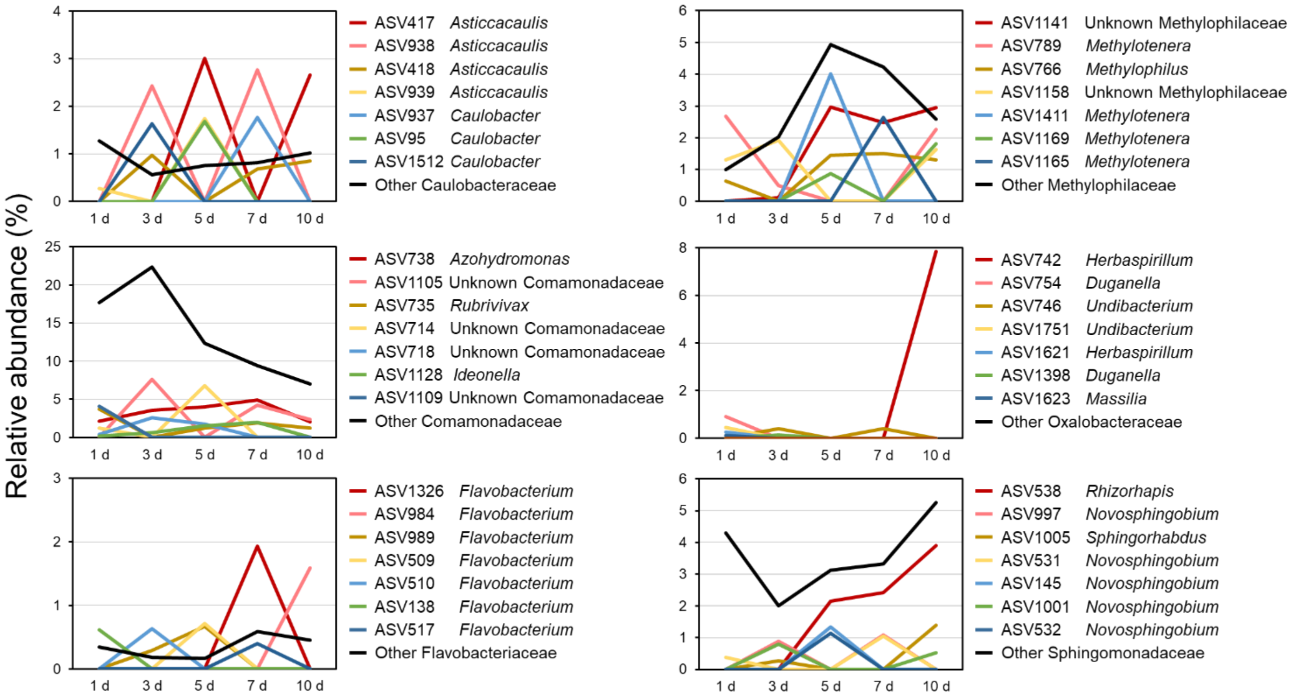

Fig. S1 Dominant ASVs during the 10-day cultivation in Inukai Pond. Relative abundances of the top seven ASVs in each of the six dominant families are shown. (A) Caulobacteraceae, (B) Comamonadaceae, (C) Flavobacteriaceae, (D) Methylophilaceae, (E) Oxalobacteraceae, (F) Sphingomonadaceae.

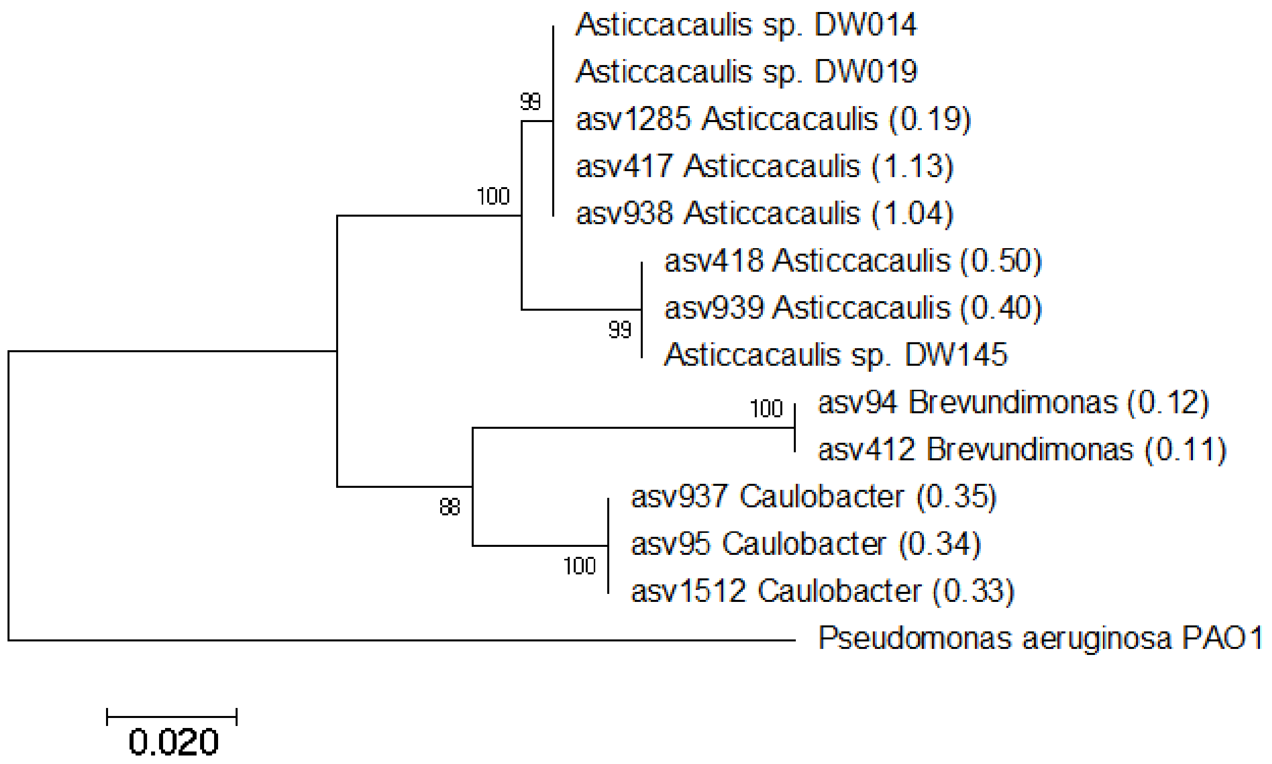

Fig. S2 Neighbor-joining tree based on partial 16S rRNA gene (255 bp) showing the phylogenetic relationship of strain DW014, DW019 and DW145 among dominant ASVs in Caulobacteraceae (> 0.1%). Values in the parentheses show the mean of relative abundance (%) in the pond-grown duckweed microbiome analyzed after 1, 3, 5, 7, and 10 days. Numbers at nodes indicate percentages of replicate trees in which the associated taxa clustered together in the 1,000 bootstrap test. *Pseudomonas aeruginosa* PAO1 (accession no. DQ777865) was used for the outgroup rooting.

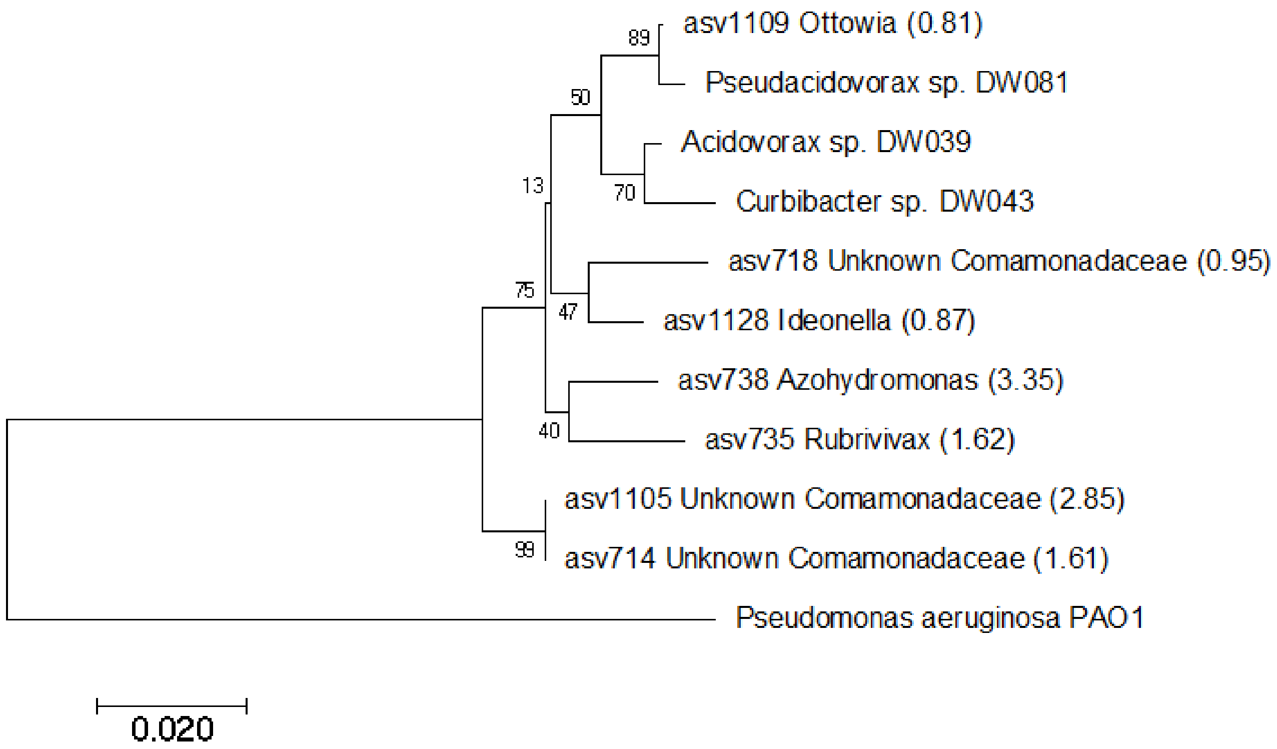

Fig. S3 Neighbor-joining tree based on partial 16S rRNA gene (258 bp) showing the phylogenetic relationship of strain DW039, DW043 and DW081 among dominant ASVs in Comamonadaceae (> 0.1%). Values in the parentheses show the mean of relative abundance (%) in the pond-grown duckweed microbiome analyzed after 1, 3, 5, 7, and 10 days. Numbers at nodes indicate percentages of replicate trees in which the associated taxa clustered together in the 1,000 bootstrap test. *Pseudomonas aeruginosa* PAO1 (accession no. DQ777865) was used for the outgroup rooting.

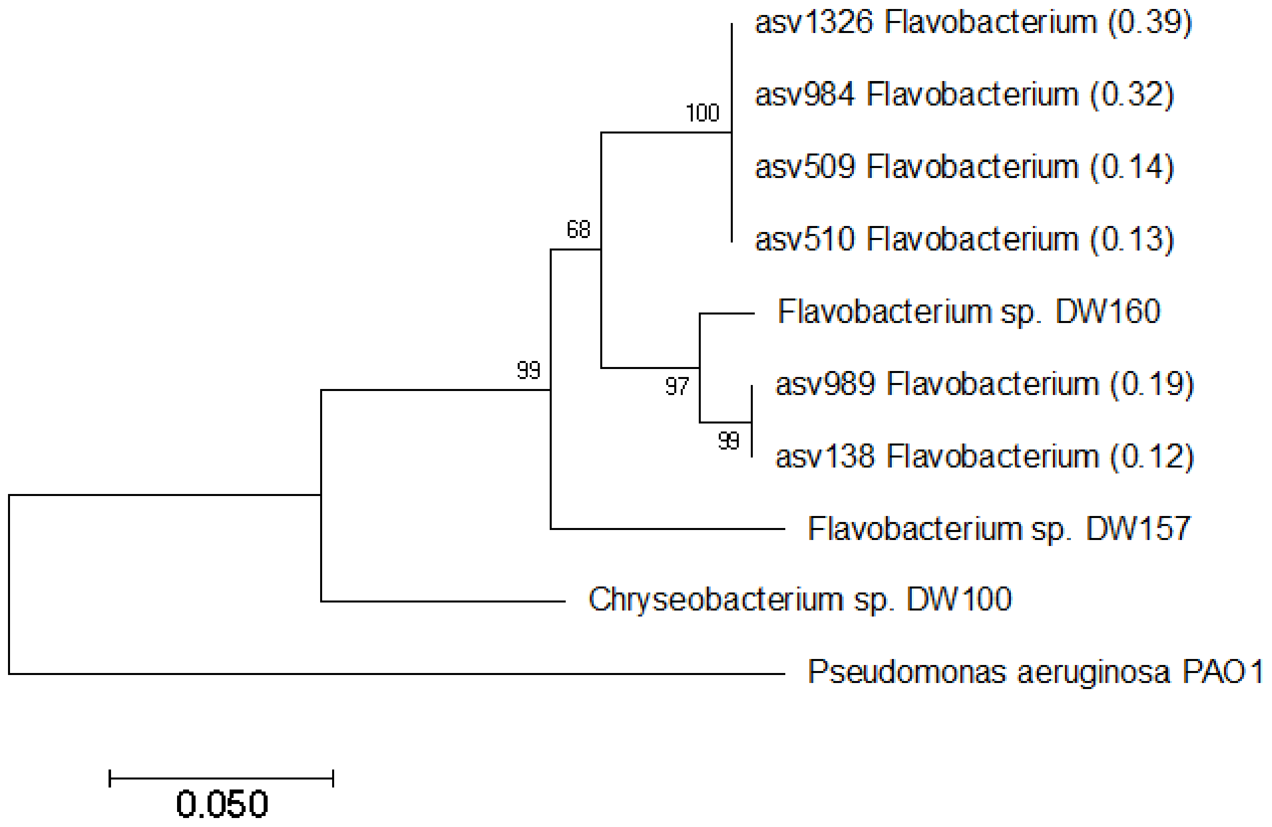

Fig. S4 Neighbor-joining tree based on partial 16S rRNA gene (255 bp) showing the phylogenetic relationship of strain DW100, DW157 and DW160 among dominant ASVs in Flavobacteriaceae (> 0.1%). Values in the parentheses show the mean of relative abundance (%) in the pond-grown duckweed microbiome analyzed after 1, 3, 5, 7, and 10 days. Numbers at nodes indicate percentages of replicate trees in which the associated taxa clustered together in the 1,000 bootstrap test. *Pseudomonas aeruginosa* PAO1 (accession no. DQ777865) was used for the outgroup rooting.

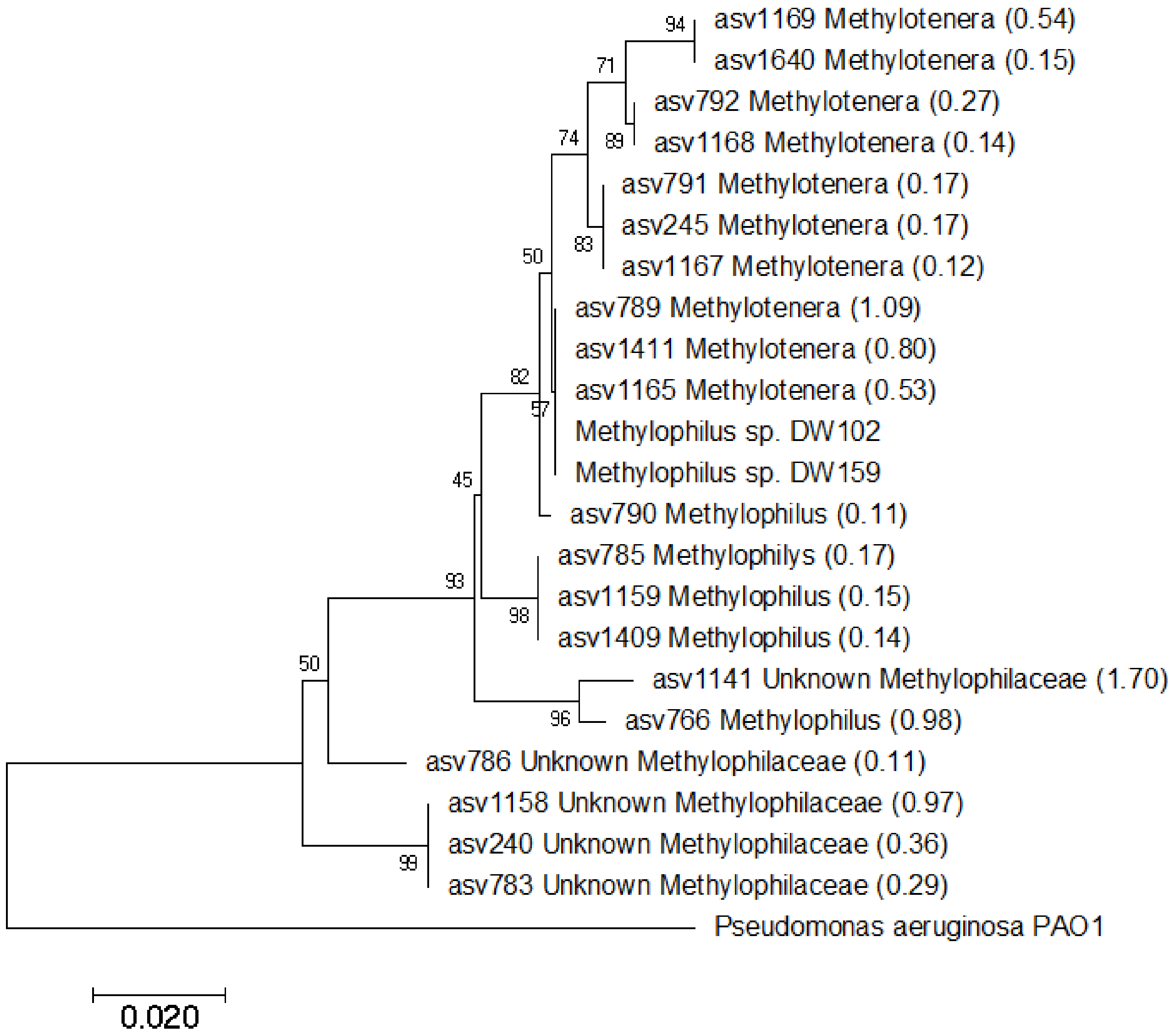

Fig. S5 Neighbor-joining tree based on partial 16S rRNA gene (256 bp) showing the phylogenetic relationship of strain DW102 and DW159 among dominant ASVs in Methylophilaceae (> 0.1%). Values in the parentheses show the mean of relative abundance (%) in the pond-grown duckweed microbiome analyzed after 1, 3, 5, 7, and 10 days. Numbers at nodes indicate percentages of replicate trees in which the associated taxa clustered together in the 1,000 bootstrap test. *Pseudomonas aeruginosa* PAO1 (accession no. DQ777865) was used for the outgroup rooting.

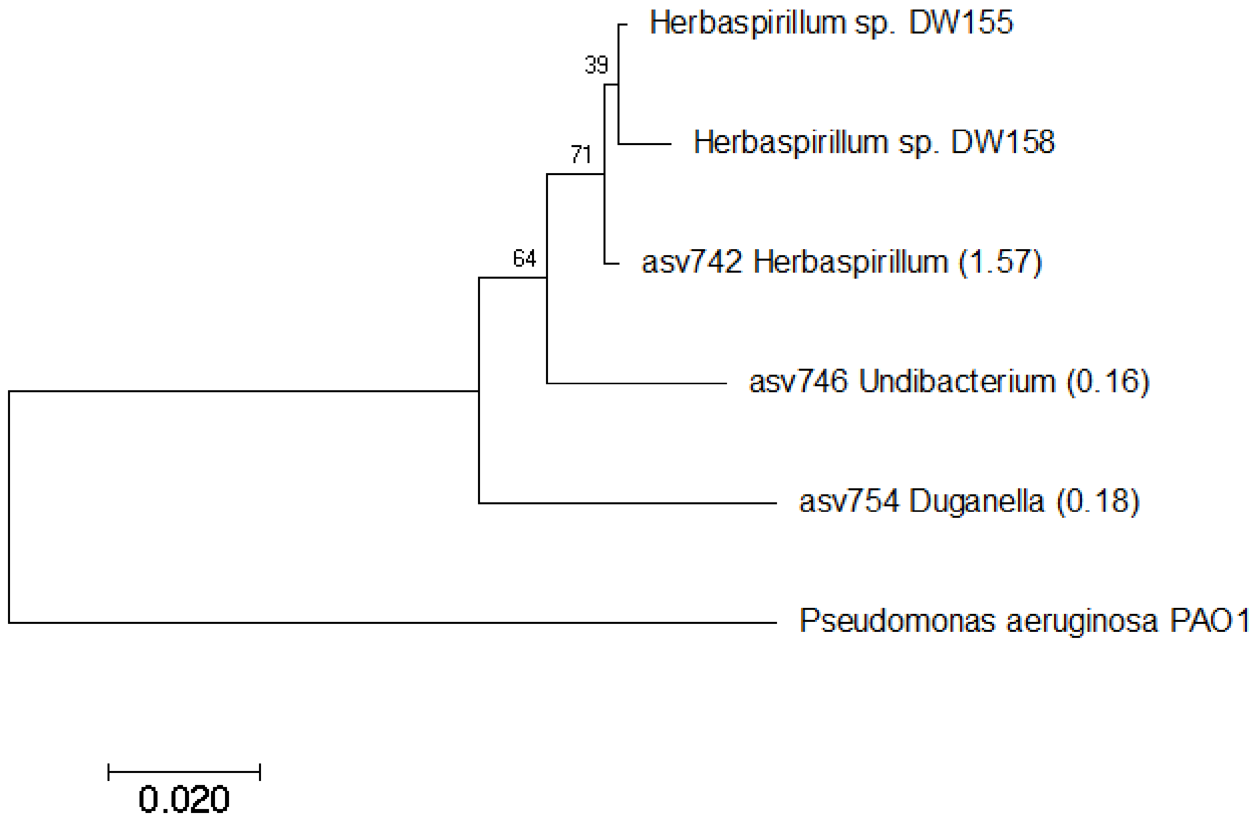

Fig. S6 Neighbor-joining tree based on partial 16S rRNA gene (260 bp) showing the phylogenetic relationship of strain DW155 and DW158 among dominant ASVs in Oxalobacteraceae (> 0.1%). Values in the parentheses show the mean of relative abundance (%) in the pond-grown duckweed microbiome analyzed after 1, 3, 5, 7, and 10 days. Numbers at nodes indicate percentages of replicate trees in which the associated taxa clustered together in the 1,000 bootstrap test. *Pseudomonas aeruginosa* PAO1 (accession no. DQ777865) was used for the outgroup rooting.

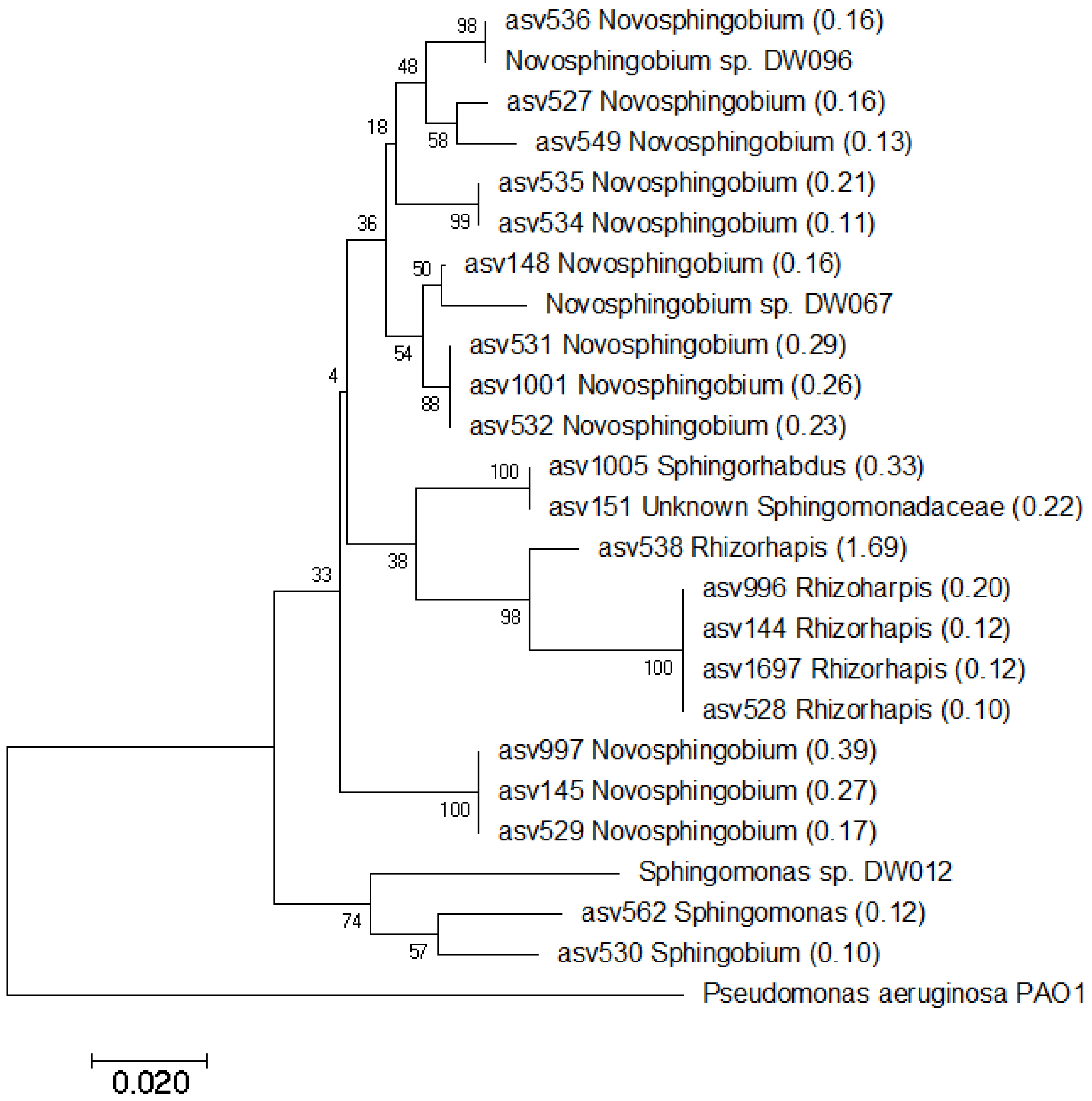

Fig. S7 Neighbor-joining tree based on partial 16S rRNA gene (257 bp) showing the phylogenetic relationship of strain DW012, DW067 and DW096 among dominant ASVs in Sphingomonadaceae (> 0.1%). Values in the parentheses show the mean of relative abundance (%) in the pond-grown duckweed microbiome analyzed after 1, 3, 5, 7, and 10 days. Numbers at nodes indicate percentages of replicate trees in which the associated taxa clustered together in the 1,000 bootstrap test. *Pseudomonas aeruginosa* PAO1 (accession no. DQ777865) was used for the outgroup rooting.
